# Human Monocytes Differentiate into Dendritic Cells Subsets that Induce Anergic and Regulatory T Cells in Sepsis

**DOI:** 10.1371/journal.pone.0047209

**Published:** 2012-10-10

**Authors:** Valérie Faivre, Anne Claire Lukaszewicz, Arnaud Alves, Dominique Charron, Didier Payen, Alain Haziot

**Affiliations:** 1 INSERM UMRS 940, Paris, France; 2 Univ Paris Diderot, Sorbonne Paris Cité, Institut Universitaire d’Hématologie, UMRS 940, Paris, France; 3 Univ Paris Diderot, Sorbonne Paris Cité, EA3509, Paris, France; 4 AP-HP, Dept of Anesthesiology and Intensive Care, Hosp Lariboisière, Paris, France; 5 AP-HP, General And Abdominal Surgery, Hosp Lariboisière, Paris, France; University of Bergen, Norway

## Abstract

**Background:**

Sepsis is a multifactorial pathology with high susceptibility to secondary infections. Innate and adaptive immunity are affected in sepsis, including monocyte deactivation.

**Methodology/Principal Findings:**

To better understand the effects of alterations in monocytes on the regulation of immune responses during sepsis, we analyzed their differentiation in dendritic cell (DC). Cells from septic patients differentiated overwhelmingly into CD1a−negative DC, a population that was only a minor subset in controls and that is so far poorly characterized. Analysis of T cell responses induced with purified CD1a−negative and CD1a+ DC indicated that (i) CD1a−negative DC from both healthy individuals and septic patients fail to induce T cell proliferation, (ii) TGFβ and IL-4 were strongly produced in mixed leukocyte reaction (MLR) with control CD1a−negative DC; reduced levels were produced with patients DC together with a slight induction of IFNγ, (iii) compared to controls, CD1a+ DC derived from septic patients induced 3-fold more Foxp3+ T cells.

**Conclusion/Significance:**

Our results indicate a strong shift in DC populations derived from septic patients’ monocytes with expanded cell subsets that induce either T cell anergy or proliferation of T cells with regulatory potential. Lower regulatory cytokines induction on a per cell basis by CD1a−negative dendritic cells from patients points however to a down regulation of immune suppressive abilities in these cells.

## Introduction

Sepsis combines an acute infection with a systemic inflammatory response syndrome, and tends to have a better prognosis due to adapted resuscitation and management measures during the first 24 hours [1]. Survivors are exposed to the risk of secondary infection [2], [3], [4] that increases length of stay in intensive care units [5], cost of care [6] and the risk of ecology changes to multi-resistant bacteria [7]. Among the factors facilitating secondary infection, immune depression induced after a first insult, including sepsis, is increasingly incriminated [8], [9], [10]. Although alterations in both innate and adaptive immune responses have been described in sepsis, the cellular and molecular mechanisms leading to increased susceptibility to secondary infections in patients have not been delineated. Current knowledge indicates that the type and intensity of adaptive responses are greatly dependent on signals delivered by the innate immune system. Analysis of innate immune responses and determining how they impact on adaptive responses in sepsis is needed to identify mechanisms that contribute to immunodepression. Several factors have been linked to immunodepression in animal models of sepsis such as inflammatory status and myeloid cell dysfunctions [11], [12], [13]. However, the relevance of these models to sepsis is uncertain [14] since immunosuppression and immunostimulation coexist [12] unlike what is found in patients.

Among alterations of innate immunity in sepsis, changes in monocyte phenotypes and functions have been extensively described with a decreased cell surface HLA-DR expression [9], [15], and impaired cytokine production to *ex vivo* stimulation [16]. Monocyte can differentiate into DC, which are endowed with pathogen sensing functions in periphery and antigen presentation to T cells in lymphoid organs. DC therefore play key roles at the interface between innate and adaptive responses and in the fitness of immune responses. Changes in the inflammatory environment can alter DC functions at all steps of their differentiation/maturation and effector functions [17]. Notably, DC with immunosuppressive functions have been described *in vivo* [18] and DC with similar properties have been produced *in vitro* by adding IL-10 during DC maturation (IL-10 DC) [19].

Alterations in adaptive immunity in sepsis include a major but transient depression of circulating lymphocyte counts due to apoptosis [20]. T cell populations are however differentially affected as CD4+CD25+CD127- regulatory T cells (Tregs) were found in higher percentage in septic patients and associated with lymphocyte anergy [21]. In addition, a predominant Th2 profile was identified in sepsis [22], [23], which may be induced by immature myeloid cells [24].

We previously analyzed monocytes from patients with septic peritonitis as well as the functions of bulk DC populations derived from these cells [25]. Circulating monocytes expressed markers of activation and/or differentiation despite their functional deactivation in responses to microbial agonists, and differentiated rapidly into DC. These DC failed however to increase their T cell activation abilities upon maturation. During the course of that previous study, we observed that a significant proportion of DCs derived from patients’ monocytes did not acquire the conventional CD1a+/CD14-negative phenotype and remained CD1a−negative. In the present study, we characterized these CD1a−negative DC, purified them, and determined their potential for functional interactions with T cells.

## Results

### Rates of CD14-negative/CD1a−negative Monocyte-derived DC in Patients and Controls

In a previous study, we have analyzed prototypical CD14-negative/CD1a+ DC generated *ex vivo* from septic patients and we found that their phenotypes (expression of HLA-DR, co-stimulatory molecules, CCR7 in steady state and after TLR activation) were similar to those of healthy controls [25]. However, a large proportion of monocytes from septic patients did not differentiate into prototypical CD14-negative/CD1a+ cells.

**Figure 1:**
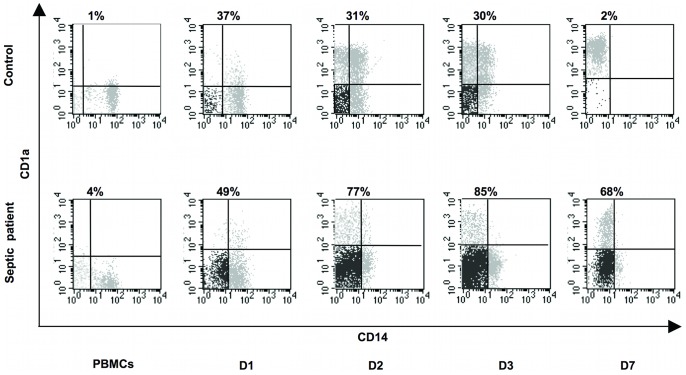
Kinetics of CD14 and CD1a expression during DC differentiation. CD14/CD1a dot plots of monocytes undergoing a 7-day differentiation into DC induced by IL-4 and GM-CSF from one control donor (upper panels) and from patient 9 (lower panels). PBMCs panels show gated monocytes before adhesion and differentiation. D1, D2, D3 and D7 dot plots show cells collected in suspension during differentiation. Quadrant markers were set according to isotype control profiles for each sample. Percentages of CD14-negative/CD1a−negative cells are given for each plot.

To characterize these cells, we studied 18 septic peritonitis patients undergoing surgery (Table 1). Analysis of CD14 and CD1a expression in monocyte-derived DC was performed at day 0, 1, 2, 3 and 7 during culture with GM-CSF and IL-4. Figure 1 shows a representative kinetic analysis for one patient and one control. Strikingly, in the patient sample a large cell population differentiated into CD1a−negative cells, resulting in a double negative CD14-negative/CD1a−negative cell population (referred as CD1a−negative) with the remainder being CD14-negative/CD1a+. As much as 68% of the cells at day 7 were CD1a−negative (Figure 1), while in cultures started from monocytes isolated from a control donor this population represented only 2% of the cells.

**Table 1:** Patients characteristics.

N°	Age	Gender	Diagnosis	SAPSII	Microorganisms in peritoneal fluid	Nb of organ failure(s)	Outcome
1	94	M	Sigmoid perforation	70	*Bacteroides fragilis, Streptococcus group D, Escherichia coli*	4	Death 1^st^ week
2	69	M	Secondary peritonitis after small intestine fistula	45	*E coli*	3	Alive
3	33	M	Primary streptococcus A peritonitis	35	*Streptococcus group A*	3	Alive
4	51	M	Peritonitis after duodenal perforation	47	*Candida albicans*	2	Alive
5	77	F	Secondary peritonitis after anastomotic small intestinefistula	44	*E coli* (abcess)	2	Alive
6	48	F	Acute necrotizing pancreatitis with infection	42	*Bacteroides fragilis, Streptococcus anginosus*	2	Death at D30
7	94	F	Gastric ulcer perforation	49	*Enterococcus faecalis, Streptococcus group C,* *Proteus vulgaris*	2	Alive
8	76	F	Stercoral peritonitis with sub-diaphragmatic abscess after lithiasis surgery and colicfistula	44	*Staphylococcus epidermidis*	1	Alive
9	89	F	Secondary peritonitis after total sigmoido-proctectomy	43	*E coli, Bacteroides fragilis*	1	Alive
10	21	M	Duonenum ulcer perforation	30	Not determined	1	Alive
11	53	M	Secondary peritonitis after trauma	31	0	1	Alive
12	39	M	Secondary peritonitis after anastomosis fistula	12	*Citrobacter koseri, E coli, Streptococcus* *intermedius*	1	Alive
13	77	M	Peritonitis after rectal perforation	47	*E coli, Klebsiella pneumoniae*	1	Alive
14	71	M	Stercoral peritonitis after sigmoid perforation	40	*E coli*	1	Alive
15	55	M	Gastric ulcer perforation	24	0	0	Alive
16	55	F	Gastric ulcer perforation	24	0	0	Alive
17	86	F	Appendicular peritonitis	24	*Streptococcus constellatus*	0	Alive
18	75	F	Secondary peritonitis after colectomy	24	*E coli, Bacteroides fragilis*	0	Alive

**Figure 2:**
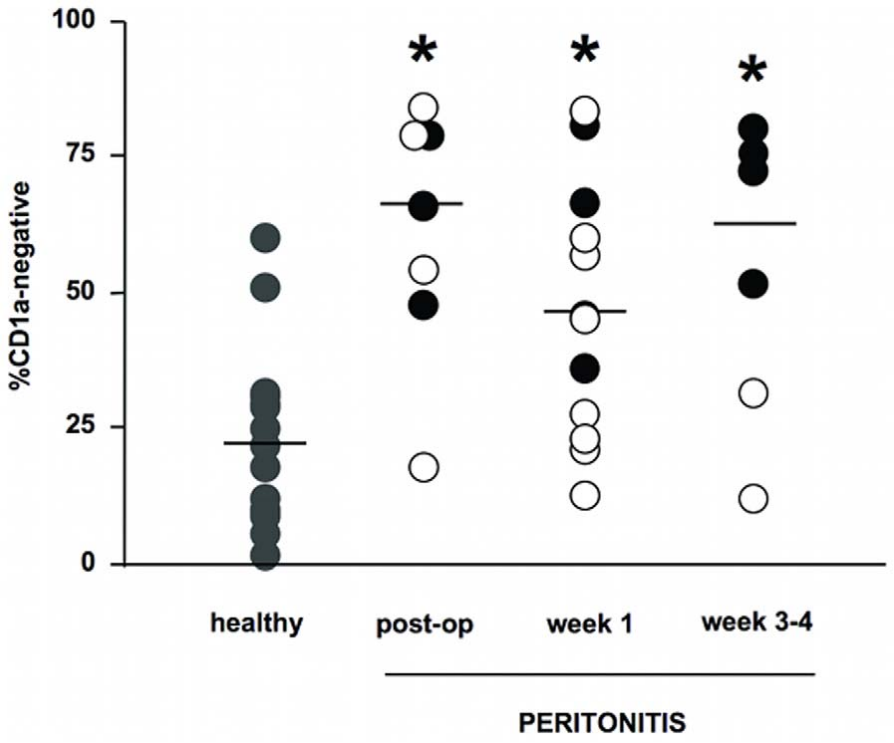
Percentages of CD1a−negative cells in 3-day cultures of differentiating monocytes. Monocytes from control donors (n = 15), post-operative (n = 7), week 1 (n = 12), and week 3–4 (n = 6) patients were differentiated into DC in the presence of IL-4 and GM-CSF. Individual values and median of each group is shown. In patients group, black circles: 2 or more organ failures at Day 0 of peritonitis; white circles: less than 2 organ failures. *, p<0.05, Mann Whitney non parametric test between controls and patients.

We looked for the presence of these CD1a−negative cells during differentiation of monocytes from blood collected from patients on the day of surgery (post-op), one week, and 3–4 weeks later. We observed that at day 3 of cell culture CD1a−negative cells were in higher proportion in most patients compared to control donors (Figure 2) (p<0.05, Mann-Whitney) and remained high over the 3–4 week period of analysis. Interestingly, patients who showed more than 50% CD1a−negative cells 3–4 weeks after surgery were those with 2 or more organ failures at inclusion time (Figure 2, black circles). Five out of 7 patient immediate post-operative samples generated more than 50% CD1a−negative cells at day 3 of culture, whereas only 2 out of 15 control samples reached this level. As much as 5 out of 12 patient samples collected 1 week after surgery still had more than 50% CD1a−negative cells, and this was still the case for 4 out of 6 patients 3–4 weeks after surgery, suggesting a long lasting functional alteration of monocytes in septic peritonitis. At day 7 of the culture, the proportion of CD1a−negative cells was also higher in patients compared to controls (p<0.05, Mann-Whitney for ‘post-op’ and ‘week 1’, NS for ‘week 3–4’, data not shown) although the difference was less marked than at day 3 of cell culture. In subsequent studies we used samples drawn one week after surgery.

The presence of CD1a−negative cells in control samples (median [IQR]: 21.6 [9.7] %), although lower than in patients, indicated that their development could also arise from control monocytes and we used these CD1a−negative cells as reference for the analysis of those derived from patients. Since CD1a is a commonly used marker for DC, we sought to confirm that these CD1a−negative cells produced in the presence of GM-CSF and IL-4 were effectively DC. We therefore checked the expression of several molecules known to be either up- or down-regulated during monocyte differentiation into DC, and during DC maturation (Table 2). Results indicated that immature CD1a−negative cells generated from control monocytes expressed cell surface molecules characteristic of conventional CD1a+ DC such as receptors involved in internalization (CD206 and DC-SIGN), in antigen presentation and costimulation (HLA-DR, CD80, and CD86). Upon activation with lipopolysaccharide (LPS) these markers were up-regulated and the DC maturation marker CD83 was expressed. In addition, CD1a−negative cells expressed low levels of Fc receptors FcgammaRII (CD32) and FcgammaRIII (CD64) primarily found on macrophages. In preliminary experiments, patient samples were similarly analyzed for differentiation and maturation of DC. Results indicated overall similar patterns of expression between control and patient CD1a−negative cells except for a stronger expression of Fc receptor CD32 in patients iDC (expression in mDC was similar) and of mannose receptor CD206 in patients mDC (expression in iDC was similar) (data not shown). Importantly, the expression of DC-SIGN, a feature of DC, was similar in control and patient CD1a−negative iDC and mDC (data not shown). Moreover, internalization of BSA and dextran in purified CD1a−negative cells were similar to conventional non-sorted DC and purified CD1a+ cells derived from control monocytes differentiated into immature DC (iDC) (at day 7 of culture) and mature DC (mDC) (after 48 hours exposure to LPS) (not shown). Maturation equally decreased internalization capacities of non-sorted, CD1a+ and CD1a−negative subsets. Together these results indicated that CD1a−negative cells were of DC lineage.

**Table 2:** Flow cytometry phenotyping of non-sorted, sorted CD1a+, and sorted CD1a−negative cells from 3 control donors.

	Non-sorted		CD1a+		CD1a−negative	
	iDC	mDC	iDC	mDC	iDC	mDC
HLA-DR	+++	+++	+++	+++	+++	+++
CD80	±	++	±	++	±	++
CD83	±	++	±	++	±	++
CD86	±	+++	±	+++	+	++
CD32	±	±	±	+	+	±
CD64	±	±	±	±	±	±
CD1b	++	++	++	++	++	++
CD206	++	++	++	++	++	±
DC SIGN	++	++	++	++	++	+

Cells were sorted at day 3 of monocyte differentiation, put back in culture for 4 additional days (iDC), and matured for 2 days with 10 ng/ml LPS (mDC).

Minimum and maximum fluorescence values (number of sites per cell) of each group in each cell type were determined and results were scored as follows:

0 site per cell: 0; low to 5 000 sites: ±; 5 000 to 10 000 sites: +; 10 000 to 100 000 sites: ++; >100 000 sites: +++.

**Figure 3:**
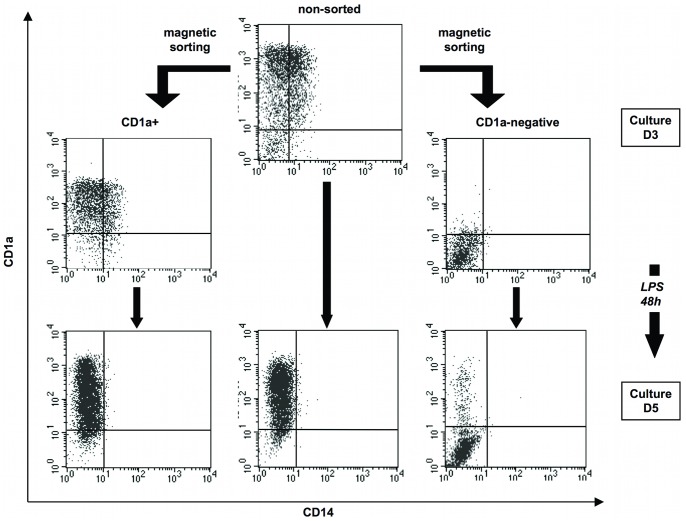
Procedure used for generation, purification and maturation of CD1a−negative and CD1a+ DC. Monocytes (here from a control donor) were cultured for 3 days in the presence of IL-4 and GM-CSF and separated by magnetic sorting into CD1a+ and CD1a−negative populations which were matured for 48 h in the presence of LPS (10 ng/ml).

To determine how maturation affected the CD1a expression in CD1a−negative DC, we analyzed its expression in monocyte differentiated into DC for 3 days, sorted into CD1a+ and CD1a−negative cell populations, followed by culture for 48 hours with or without LPS used as a maturation agent. Figure 3 shows one representative experiment analyzing CD14 and CD1a expression in control cells, indicating that a large majority (85% in this sample) of CD1a−negative cells generated at day 3 did not increase their CD1a expression during an additional 48-hour culture in the presence of LPS (Figure 3, right panels) or in its absence (not shown). A similar evolution was observed with patients samples (not shown) showing the stability of this cell phenotype. Interestingly, more cells remained CD1a−negative at day five in the sorted CD1a−negative fraction than in the bulk non-sorted population, suggesting cooperative effects in mixed cultures leading to CD1a expression.

### Expression of Cytokines in CD1a+ and CD1a−negative Cells from Patients and Controls

**Figure 4:**
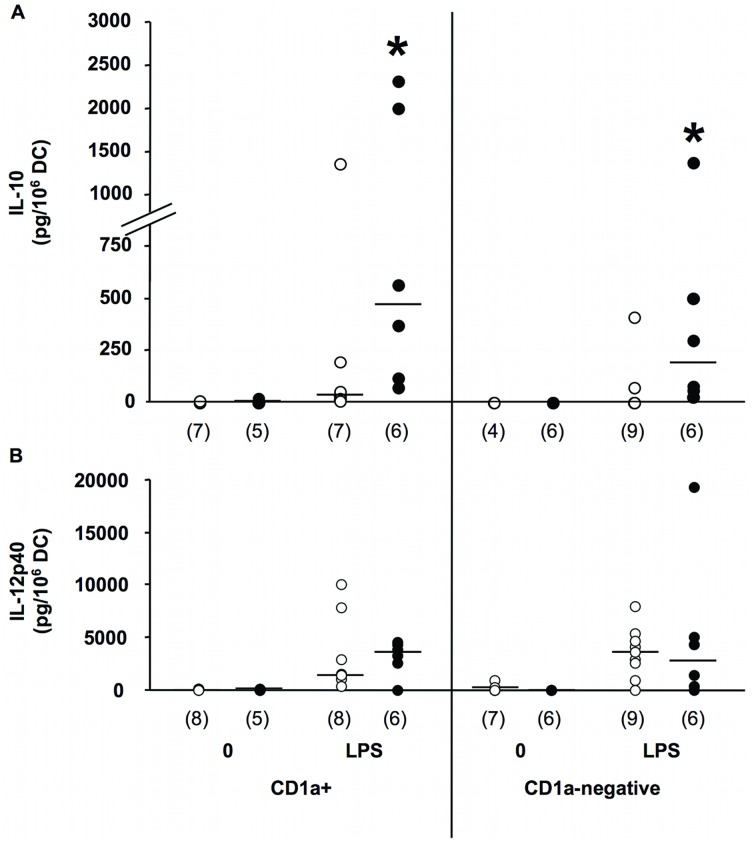
Cytokine production by CD1a+ and CD1a−negative DC derived from septic patients. IL-10 (A) and IL-12p40 (B) levels were assayed in culture supernatants of CD1a+ and CD1a−negative DC purified at day 3 of monocyte differentiation and cultured an additional 48 h in the absence (0) or the presence of LPS (10 ng/ml) for maturation (LPS). Results are shown as individual values with median for each group. Patients, filled circles; controls, opened circles. Numbers (n) in each group are indicated. *, p<0.05, Mann Whitney non parametric test between controls and patients.

To further characterize these CD1a+ and CD1a−negative DC populations, we determined their production of IL-10 and IL-12p40 as immature DC and after maturation with LPS. IL-10 production in immature CD1a+ and CD1a−negative DC from patients and control donors was similar and was low or negative (Figure 4A). In contrast, both mature CD1a+ and CD1a−negative DC produced IL-10 and this LPS-induced release was significantly higher in patients cells (p = 0.0321), suggesting an anti-inflammatory orientation of both types of DC in patient samples. Similarly to IL-10, IL12p40 was low in immature cells. Its induced expression was however similar in patients and control cells of CD1a+ and CD1a−negative phenotype (Figure 4B).

### Antigen Presentation by CD1a+ and CD1a−negative Cells from Patients and Controls

**Figure 5:**
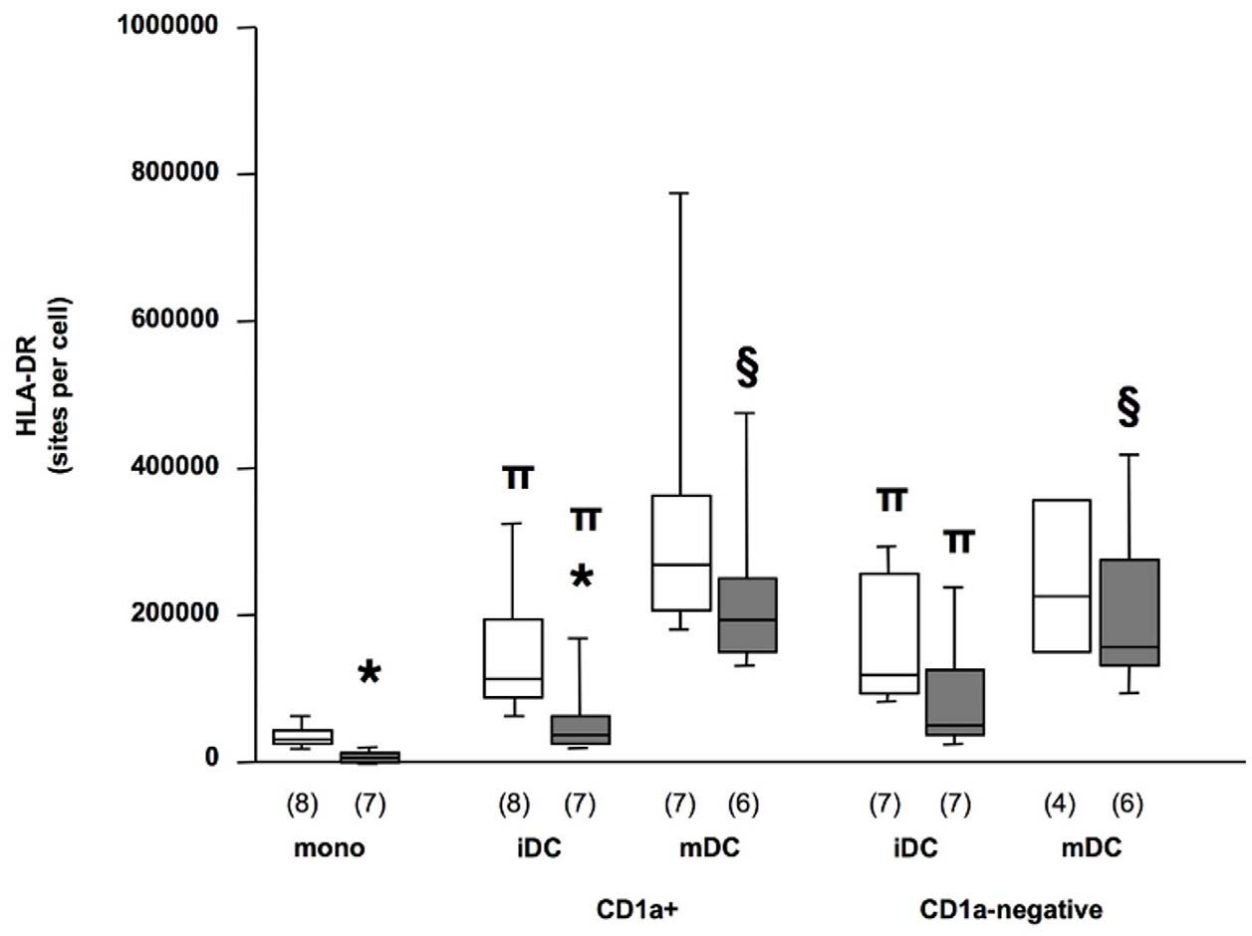
HLA-DR surface expression in CD1a+ and CD1a−negative DC derived from septic patients. Flow cytometry analysis of HLA-DR in monocytes, and in CD1a+ and CD1a−negative DC purified at day 3 of monocyte differentiation (immature DC, iDC) and cultured an additional 48 h in the presence of LPS at 10 ng/ml (mature DC, mDC). Patients, filled boxes; controls, opened boxes. Numbers (n) in each group are indicated. Box plot represent median ±25^th^–75^th^ percentile. * p<0.05 Mann Whitney test between controls and patients, ∏ p = 0.018 and p = 0.0117 in patients and controls respectively, Wilcoxon test between mono and iDC CD1a+; p = 0.018 both in patients and controls, Wilcoxon test between mono and iDC CD1a−negative, § p<0.05 Wilcoxon test between iDC and mDC.

To explore antigen-presenting capacities of CD1a+ and CD1a−negative DC, we first analyzed their HLA-DR expression (Figure 5). As expected [15], [25], monocytes HLA-DR was lower in patients than in control donors (p = 0.0018). Although its expression significantly increased after 3 days of differentiation (p<0.02 for iDC CD1a+ vs. monocytes and for iDC CD1a−negative vs. monocytes, for both patients and controls) a significant difference persisted between patient and control iDC of CD1a+ phenotype (p = 0.0206). The HLA-DR expression in CD1a−negative iDC from patients was also reduced compared to control CD1a−negative cells although the difference was less pronounced and not significant. Maturation induced a strong increase of HLA-DR expression in both CD1a+ and CD1a−negative DC from patients (p = 0.0277 for iDC vs. mDC in both CD1a+ and CD1a−negative DC) and this up-regulation was stronger than in control cells, resulting in similar level of expression in mature CD1a+ and CD1a−negative DC from patients and controls. In addition, these results indicated that maturation induced after 3 days of differentiation strongly up-regulated the expression of HLA-DR, a major marker of DC maturation, on CD1a+ and CD1a−negative DC, although other markers of maturation (CD80, CD86, CD83) were only tested after a 7-day differentiation period (Table 2).

**Figure 6:**
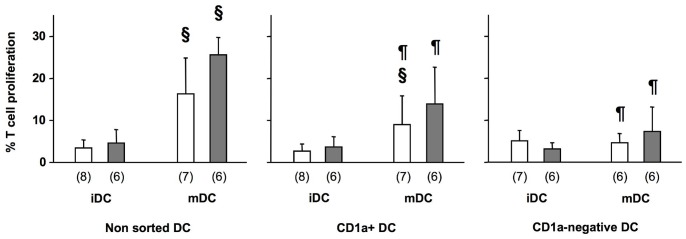
Allogenic T cell proliferation induced by DC derived from septic patients. MLR induced by non-sorted, CD1a+, and CD1a−negative DC purified at day 3 of monocyte differentiation (immature DC, iDC) and cultured an additional 48 h in the presence of LPS at 10 ng/ml (mature DC, mDC). The percentage of proliferation of CFSE-labeled T cells was determined as described in Methods. Patients, filled bars; controls, opened bars. Numbers (n) in each group are indicated. Histograms represent mean ± SD. § p<0.05 Wilcoxon non parametric test between iDC and mDC, 2 p<0.05 Wilcoxon non parametric test between non-sorted and either CD1a+ or CD1a−negative mDC.

To determine the capacity of each of these DC populations to activate T cells, we analyzed the proliferation of T cells that they induced in MLR. As expected, T cell proliferation was significantly higher with mDC compared to iDC using bulk non-sorted DC population from control donors (Figure 6) an effect also found with mDC from patients (Figure 6, p = 0.018 and p = 0.0277 respectively). However, purified CD1a+ and CD1a−negative DC populations showed different abilities in MLR. In patients and controls, CD1a+ and CD1a−negative mDC induced a lower T cell response than non-sorted mDC (p<0.05). Even so, control CD1a+ mDC induced a significantly increased T cell proliferation compared to corresponding iDC (p = 0.018). In contrast, increased proliferation induced by CD1a+ mDC from patients did not reach statistical significance. Contrarily to CD1a+, no increase in T cell proliferation was observed with mature CD1a−negative DC derived from control donors compared to iDC. A slight, although non significant increase in T cell proliferation was found with CD1a−negative DC from patients that may indicate a very small enhancement of T cell activation abilities compared to their control counterpart. This low responsiveness was not the result of insufficient HLA mismatch since in each MLR experiment healthy donor and patient total, unfractionated DC were co-cultured with the corresponding allogenic T cells and none of these controls was negative. Together, these results showed that the CD1a−negative DC populationwas unable to induce T cell proliferation possibly generating anergic T cells. In addition, T cell proliferation induced by sorted CD1a+ cells was significantly lower than proliferation induced by corresponding bulk DC, suggesting either an interaction between CD1a+ and CD1a−negative cells to promote T cell responses or the loss/modification of a DC population during cell purification.

### Foxp3 Expression in T Cells after MLR

**Figure 7:**
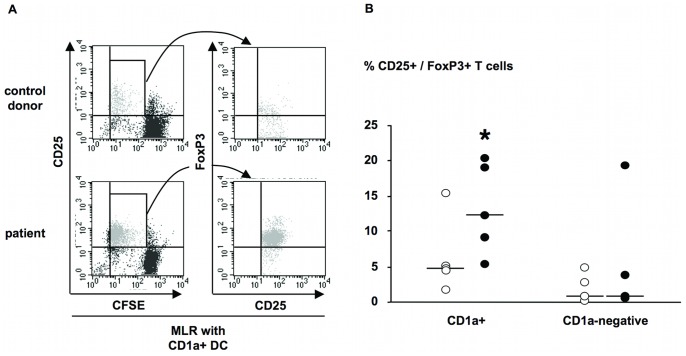
FoxP3 expression in CD25+ proliferating T cells after MLR DC derived from septic patients. MLR was induced by CD1a+ and CD1a−negative DC purified at day 3 of monocyte differentiation and cultured an additional 48 h in the presence of LPS (10 ng/ml) for maturation. A, representative FACS dot plot of T cells after MLR with CD1a+ mature DC from one control donor and patient 4, analyzed for surface CD25, CFSE label, and Foxp3 intracellular expression. Foxp3 expression was analyzed on gated CD25+ and proliferating cells according to CFSE label. B, percentage of CD25+/Foxp3+ T cells after MLR induced by CD1a+ and CD1a−negative mature DC. Results are shown as individual values with median for each group. Patients, filled circles (n = 5); controls, opened circles (n = 5). *, p<0.05 Mann Whitney test between control donors and patients.

To further characterize the functional properties of these DC populations, we analyzed the phenotype of T cells after MLR. Immune responses in septic patients are depressed, and T cells with regulatory functions may be involved in this immunodepression. Although no specific marker for this T cell subset is yet available, the transcription factor Foxp3 is expressed in subsets of Tregs and comparing its expression induced in T cells cultured with patient or control DC is informative. We therefore assessed Foxp3 expression in T cells co-cultured with DC derived from patient and control monocytes. As shown with representative samples in figure 7A left panels, a large proportion of T cells that proliferated after co-culture with CD1a+ DC from patients or controls were CD25+. However, when these cells were analyzed for their expression of Foxp3, 93% of proliferating CD25+ T cells cultured with patient CD1a+ DC expressed high levels of Foxp3 (Figure 7A, right panels) compared to 40% of Foxp3 T cells induced by control CD1a+ DC in the corresponding T cell population. Figure 7B indicates the percentage of CD25+Foxp3+ T cells after MLR for groups of patients and control donors. The proportion of CD25+Foxp3+ T was more than 2-fold higher with T cells co-cultured with CD1a+ DC from patients compared to control donors. In contrast, in T cells cultured with CD1a−negative DC from patients or controls, CD25+Foxp3+ T cell percentages were similar and low. These results suggest that CD1a+ DC from septic patients induce the proliferation of T cells with a stronger potential for regulatory functions than in control conditions.

### Cytokine Production Induced by CD1a+ and CD1a−negative DC in MLR

**Figure 8:**
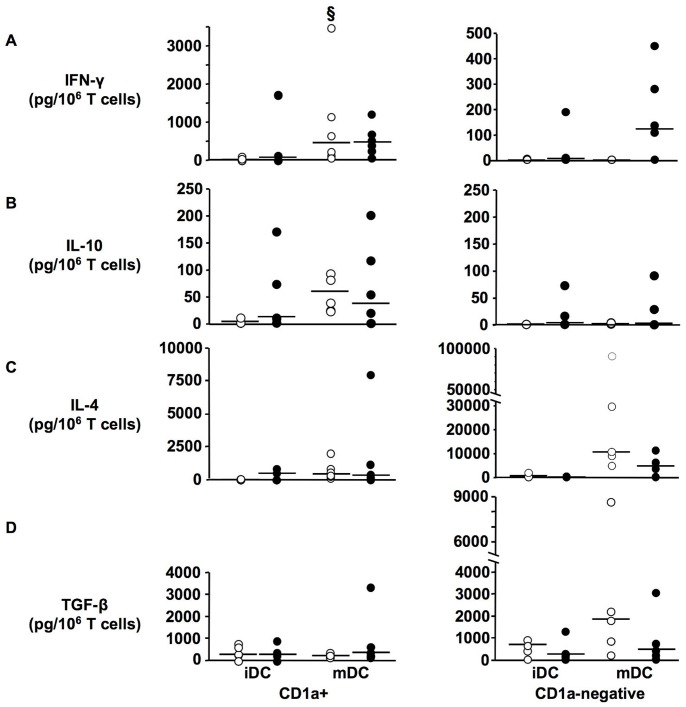
Cytokine production induced by DC derived from septic patients in MLR. MLR was induced by CD1a+ and CD1a−negative DC purified at day 3 of monocyte differentiation (immature DC, iDC) and cultured an additional 48 h in the presence of LPS at 10 ng/ml (mature DC, mDC). Concentrations of IFN-γ (A), IL-10 (B), IL-4 (C), and TGF-β (D) were assayed in cultured supernatants and expressed in picograms per millions T cells initially present in cultures. Results are shown as individual values with median for each group. Patients, filled circles (n = 6); controls, opened circles (n = 6 except CD1a−negative iDC and mDC n = 5). § p<0.05 Wilcoxon non parametric test between iDC and mDC.

Cytokines known to reflect Th1, Th2 or regulatory polarization were measured in MLR supernatants. Mature CD1a+ DC from controls induced a strong IFN-γ secretion (p = 0.0277, iDC vs. mDC) and the corresponding cells from most of the septic patients also induced IFN-γ secretion although as a whole the difference was not significant compared to iDC (Figure 8A, left panel). Limited amounts of IL-10 were induced by mature CD1a+ DC from patients with no difference with controls (Figure 8B, left panel). IL-4 production was low and its amounts were actually similar with immature and mature CD1a+ DC from patients (Figure 8C, left panel). TGF-β was low in all MLRs using CD1a+ cells (Figure 8D, left panel). Together, these results are consistent with a Th1-like profile for T cells activated with CD1a+ DC although less pronounced with patient cells.

On the contrary, MLR cytokines induced by CD1a−negative DC differed strongly from those induced by CD1a+ cells and were also distinct between control and patient cells. Although individual variations were sizeable, most samples from control donors mature CD1a−negative DC induced very large amounts of IL-4 and TGF-β during MLR (though not statistically significant, p = 0.0679 for both cytokines, iDC vs. mDC) whereas IFN-γ and IL-10 were not induced (Figure 8, right panels). In contrast, CD1a−negative DC from patients induced lower amounts of IL-4 and especially of TGF-β although the difference between controls and patients was not statistically significant. Interestingly, these cells also induced a low production of IFN-γ, with levels around 100 pg/ml in 4 out of 6 patients (Figure 8, right panels), whereas it was undetectable in all control samples (p = 0.0679). IL-10 was low or not detectable in most MLRs with CD1a−negative DC. Together these results indicated that the minor CD1a−negative DC derived from control monocytes constitute a unique population of DC with likely regulatory and/or Th2 inducing functions. In patient samples, this population which is abundant appeared to have a more complex profile with lower observed regulatory/Th2 cytokine induction abilities and a trend to a unique low induction of IFN-γ.

## Discussion

During sepsis immune responses associate intricate hyperactivation and depression differing in type and intensity during disease evolution and in distinct anatomical compartments [8], [26]. In patients surviving the initial days of pro-inflammation, susceptibility to secondary infection becomes a major risk [2], [9] and disruption of the delicate balance between anti-infection pro-inflammatory responses and compensatory anti-inflammatory responses may favor this susceptibility. To investigate the mechanisms through which sepsis-induced immunodepression may contribute to susceptibility to infection, we examined the functions of DC derived from monocyte precursors in patients. We showed that circulating monocyte from peritonitis patients differentiated into CD1a−negative DC in a much larger proportion than control monocytes. With control donors, this population of DC was much smaller and, when purified and co-cultured with allogenic T cells, it tended to induce the production of large amounts of TGF-β and IL-4, indicating the induction of regulatory properties in T cells that remained Foxp3-negative. By contrast, sepsis CD1a−negative DC induced less IL-4 and TGF-β in MLR cultures. They showed a trend to generate a low but consistently detected IFN-γ production, suggesting overall reduced regulatory abilities and the emergence of a mild pro-inflammatory potential in this DC subset from patients. TGF-β is the major inducer of as well as product from Th3 cells, a subset of Tregs [27], and some Th3 clones were able to produce IL-4 [28]. T cells co-cultured with CD1a−negative DC shared these characteristics. Th3 cells are also able to induce the expression of Foxp3 and suppressive functions in CD25+ and CD25-negative T cells in a TGF-β-dependent mechanism [27]. This effect may however be countered by high amounts of IL-4 which inhibits Foxp3 expression [29]. In any case, these T cells would antagonize Th1 responses. Importantly, this potential for Th1 antagonizing effects was not as strong in cultures from septic patients. In addition to the apparent but not significant decreased induction of anti-inflammatory cytokines, CD1a−negative DC from septic patients tended to induce IFN-γ production in the context of sepsis where anti-inflammation predominates with high serum IL-10 [25]. This T cell cytokine switch to low TGF-β/IL-4 with emergent IFN-γ induced by patients CD1a−negative DC, together with the enhanced development of this DC population in septic conditions, might help build up immunity following injury-induced immunodepression.

Whereas CD1a−negative DC from control donors and patients intricately differed in the cytokine profiles they induced in T cells, they shared a major defect in inducing a significant T cell proliferation in vitro, an effect that could be related to TGF-β production [30]. In contrast, lymphoproliferation was induced by CD1a+ DC in both septic and control groups although the difference between mDC and iDC in sepsis did not reach statistical significance. However, CD1a+ DC derived from sepsis and controls differed strongly with respect to Foxp3 induction in proliferating T cells with a two- to three-fold stronger Foxp3 expression in CD25+ T cells activated by patients DC. This raises the possibility that this DC subset could activate natural Tregs or elicit inducible Tregs in septic patients. Still, T cell-induced cytokine profiles were similar when sepsis and control CD1a+ DC were used, with comparable production of IL-10 and IFN-γ. Activated natural Tregs have been shown to be low producers of cytokines [31]. The identification and delineation of Tregs subsets using markers and profiles of cytokine expression remain arduous, and functional testing of suppressive activity was not possible here given the small size of samples obtained from patients in critical condition. The suppressive nature of human Foxp3+ T cell generated *in vitro* has been controversial. Presence or absence of suppressive activity could be explained by the use of different suppression protocols, particularly the lack of enrichment in Foxp3+ T cells in assays that found no suppression [32]. Moreover, high levels of Foxp3 expression, as opposed to low or moderate expression, is linked to T cells with outright or potential to develop regulatory functions [33]. In results presented here, given the strong level of expression of Foxp3 found in highly dividing CFSE-low T cells and the lack of change in the cytokine profile between control Foxp3-low and sepsis Foxp3-high T cell cultures, our results are compatible with an expansion of natural Tregs in the presence of CD1a+ DC derived from septic patients. In sepsis, regulatory effects due to Foxp3+ T cells combined to an abundance of precursors for CD1a−negative DC that induce T cell anergy with production, albeit reduced, of regulatory cytokines, would likely contribute to the immunodepression described in patients. Recently, the expression of T cell regulatory markers and of their corresponding ligands on antigen presenting cells [34] have been found in postmortem tissues of patients that died of severe sepsis, suggesting a broad regulatory orientation of immune responses affecting innate and adaptive immunity in human sepsis.

Endotoxemia and sepsis induce alterations in bone marrow cells and their release in blood [24], [35], leading to circulating myeloid progenitors and immature myeloid cells, collectively identified as myeloid-derived suppressor cells (MDSC). Phenotypic characterization of human MDSC is still tentative [36]. In this study, we restricted the analysis of DC differentiation to mature circulating monocytes isolated by plastic adherence as this procedure negatively selects bone marrow progenitors, albeit rare primitive hematopoietic stem cells may adhere [37]. This excludes the possibility that high numbers of CD1a−negative DC would be derived from immature cells. Since these immature cells have suppressive activity [38], it is expected that in sepsis their functions would enhance the regulatory responses induced by monocyte-derived cells as described here.

Sepsis is one of several severe injuries were a depression of immune responses has been documented. Similar signs of immunodepression, such as hypo-reactivity and decreased expression of HLA-DR in monocytes, have been described in patients with trauma, extensive burns, and stroke [10]. These acute stresses could play a major role in orienting the immune responses. Data presented here in septic patients with amplified populations of CD1a−negative and regulatory CD1a+ DC may therefore result from the combined effects of infection and stress. However, attempts at comparing immune and inflammatory parameters in septic and non-septic critical care patients suggested that amplitude and duration of shared immuno-inflammatory alterations rather than pre-existing infection determined the severity status and high susceptibility to secondary infection [9], [39]. Further work will determine if other categories of critically ill patients present similar alterations in differentiated monocytes. This could be expected since the DC alterations that we observed were found in patients with heterogenous SAPSII profiles, organ failure numbers, and microorganisms in the peritoneal fluid.

In this study, patient and control cells were differentiated in standard culture conditions. Therefore, alterations in differentiation of patients’ cells indicate intrinsic modifications in these cells. The mechanisms underlying this durable commitment to regulatory responses may result from exposure to various pro- and anti-inflammatory mediators *in vivo*. The use of standard culture conditions allowed to analyze cell differentiation potential independently of patient plasma environment, which can include very heterogenous levels of inflammatory mediators as we have shown [25]. Interestingly, we found no relation between plasma IL-10 concentrations in patients and the corresponding proportion of CD1a−negative DC obtained in culture (not shown).

CD1a was used to follow monocyte differentiation into DC since it is identified as a prototypical DC marker [40]. However, some DC subsets have been described with no CD1a expression after *in vitro* differentiation of control monocyte and CD34+ progenitors [41], [42]. These were considered possible *in vitro* counterparts to dermal DC and higher cell numbers were required to induce lymphocyte proliferation compared to CD1a+ DC [42]. These CD1a−negative DC were lower producers of IL-12p70 and lower Th1 inducers than the CD1a+ cells, without inducing Th2 polarization [41]. Here, we confirmed and extended these observations using control monocytes showing that CD1a−negative DC actually induced no T cell proliferation but led to high IL-4 and TGF-beta production in MLR. Numerous differences in experimental conditions (monocyte purification, differentiation protocol, T cell responses analysis) may account for these discrepancies. The generation of CD1a−negative DC from monocytes of control donors suggests that monocyte populations from which this particular type of DC arises are present at variable rates in apparently healthy individuals and are consistently increased in sepsis. It remains to determine if increased CD1a−negative DC from control monocytes may be transient only, contrarily to increases in patients which were found over a period of 1 to 3 weeks.

Together, our results identified a skewed development of monocyte populations with regulatory potential in sepsis possibly contributing to immunosuppression. Emerging slight Th1 responses induced by abundant CD1a−negative DC generated from septic patients may counterbalance immunodepression. Mechanisms leading to this phenotype may depend on strong anti-inflammatory conditions repeatedly found in septic patients. This may shift monocyte differentiation and/or functions to increased commitment to GM-CSF/IL-4-dependent differentiation into populations of dendritic cells with various regulatory potentials on T cells (induction of Foxp3 expression for CD1a+ DC, trend to skew cytokine production to Th2/Treg profiles for CD1a−negative DC). This progression from anti-inflammation to regulatory adaptive T cell responses is however mitigated by induction of a low production of IFN-γ which may represent a subsequent regulatory homeostatic and pro-inflammatory step.

## Materials and Methods

### Patients

The study was approved by the Assistance Publique Hôpitaux de Paris Cochin Hospital Ethics Committee (3 CCPPRB 2061). In this study, blood collected for routine biological examinations was used after completion of these examinations. Patients or their next of kin received information about the study and gave verbal consent for the use of blood remainders.

However, the need for informed consent was waived by the Ethics Committee. Neither cells nor plasma were stored after the end of experiments and all data was de-identified and analyzed anonymously. Eighteen patients undergoing surgery for septic peritonitis were enrolled. Patient’s clinical characteristics are summarized in Table 1. At inclusion time, organ failures were quoted using Logistic Organ Dysfunction Score (LODS) criteria as described [25]. In addition, patients severity was characterized using the standard clinical scoring systems Simplified Acute Physiology Score (SAPS II) quoted at day 0, which includes co-morbidity, biological, and functional parameters as described [25].

A first post-operative blood sample (D0) was drawn 6 h51±4 h10 (mean ± SD) after the end of surgery, and 12 h06±4 h16 after the onset of the first organ failure when present, in 11 patients. Second (week 1) and last (week 3–4) blood samples were drawn 7±1 days (n = 16) and 26±3 days (n = 6) after surgery.

Patients cells were analyzed either in 7-day (10 post-op samples, 9 ‘week 1’ samples, 6 ‘week 3–4’ samples) or in 3-day (7 post-op samples, 12 ‘week 1’ samples, 6 ‘week 3–4’ samples) differentiation protocols.

Fifteen healthy donors were used as controls.

### Blood Sample and Cell Separation

Blood collection, peripheral blood mononuclear cells (PBMC) separation, monocyte purification and differentiation in DC, LPS-induced DC maturation, and T cell separation were performed as previously described [25].

In an alternate protocol, non-adherent cells from patients week 1 samples, and control donors samples were harvested at day 3, centrifuged, and the medium was stored at 37°C for culture continuation. Cells were washed, resuspended in cold separation buffer (phosphate buffer saline (PBS), 0.5% bovine serum albumin (BSA), 2 mM EDTA), and incubated with 5 µg/ml biotinylated anti-CD14 (Caltag), and anti-CD1a (Biolegend) for 15 minutes on ice. After washing in cold separation buffer, anti-biotin magnetic microbeads (Miltenyi) were added for 15 minutes at 4°C. Cells were washed, resuspended in separation buffer, and submitted to magnetic separation. The negative fraction, i.e. CD14-negative/CD1a−negative (CD1a−negative) cells, was collected, then the positive fraction, i.e. cells CD14+ and/or CD1a+ (CD1a+), was eluted. Both fractions were centrifuged, and resuspended in previously stored complete RPMI medium. Non-sorted and sorted cells were cultured either for immediate maturation during 48 hours, or, for immunotyping purpose (Table 2), further differentiated for 4 days followed by maturation.

### Immunophenotyping

Cell surface markers were analyzed by flow cytometry with double or triple surface immunostaining, with the following antibodies: anti-CD14-FiTC, anti-CD1a-PC5, anti-CD83-PE, anti-CD32-PE, anti-CD64-PE, anti-CD25-PC5 (Beckman-Coulter), anti-HLA-DR-PE, anti-CD3-PerCP, anti DC-SIGN-PE, anti-CD206-PE (BD), anti-CD80-PE, anti-CD86-PE, (eBiosciences), anti-CD1b-PE (R&D).

Expression of HLA-DR, CD80, CD83, CD86, CD32, CD64, CD1b, CD206 and DC SIGN was quantified in number of sites per cell (Quantibrite™, BD).

### T Cell Separation and Mixed Leucocytes Reaction (MLR)

Healthy donors’ blood obtained from residual sample after cytapheresis was used for peripheral blood mononuclear cells (PBMC) separation by density-gradient centrifugation (Eurobio). Cell viability was assessed by Trypan blue. Pan T cells were separated by magnetic depletion (Miltenyi Biotec) according to manufacturer recommendations and frozen.

For Mixed Leucocytes Reaction (MLR), frozen T cells were thawed and washed with complete RPMI medium. Viability was assessed by Trypan blue. T cells were labeled with 5 µM Carboxyfluorescein diacetate (Invitrogen Molecular Probes) for 10 minutes at 37°C, washed with complete RPMI medium, and cultured with monocytes derived dendritic cells harvested at D3 (iDC) or after 48 h additional LPS (10 ng.ml^−1^) stimulation (mDC). DC – T cell were co-cultured at 1/10 ratio, with 1 to 5.10^4^ DC for 1 to 5.10^5^ T cells per ml. Cells were harvested after 5 days. Surface staining with anti-CD3 and anti-CD25, and intracellular staining with anti-Foxp3 (eBiosciences) were performed for flow cytometry analysis. CD3 expression was checked in cells gated for the analysis of FL1 signal, obtained from CFSE intracellular esterification. T cells division induced the decrease of intracellular content of CFDA-SE, resulting in a multiple-peak signal. The peak with the highest Mean Fluorescence Intensity (MFI) characterized cells that did not undergo division, nor decrease of CFDA-SE content, and matched the peak of labeled cells in control T cell cultures with no DC added. Following peaks with lower MFI characterized dividing cells.

Percentage of T cell proliferation was calculated as:

(all events – number of events for highest peak)*100/all events [25].

### Cytokines

ELISA kits for IL-10, IL-4 and TGF-β (optEIA™, BD) were used. Custom ELISA for IL-12p40 and IFN-γ with capture (R&D) and detection biotinylated anti-human MAb (R&D, and Thermo Fisher Scientific, respectively), and recombinant human standards (BD, and Thermo Fisher Scientific, respectively) were used. Plates were read at 450 nm (EL800, Bio-tek Instruments). Detection thresholds were (in pg/ml) 4.45±3.17 for IL-10, 9.92±7.64 for IL-4, 130.93±80.39 for TGF-β, 23.57±16.69 for IL-12p40, and 7.41±8.28 for IFN-γ.

### Statistical Analysis

Differences between control donors and patients were tested by non-parametric Mann-Whitney tests. Differences between monocytes, iDC and mDC, and between non-sorted, CD1a+ and CD1a−negative DC were tested using non-parametric Kruskal-Wallis tests. If the latter were significant, two-by-two paired comparisons were analyzed using Wilcoxon tests. Differences between mDC and cells without LPS maturation were also tested using the Wilcoxon test for paired data.
